# Pharmacists’ readiness and willingness to vaccinate the public in United Arab Emirates community pharmacies: A cross-sectional study

**DOI:** 10.12688/f1000research.131153.2

**Published:** 2024-04-22

**Authors:** Dixon Thomas, Amal Abdalla, Saeed Hussein, Jean Joury, Amin Elshamy, Sherief Khalifa, Ziad Saleh

**Affiliations:** 1 College of Pharmacy, Gulf Medical University, Ajman, United Arab Emirates; 2 GIG Gulf, Dubai, United Arab Emirates; 3 Global Medical Solutions, Abu Dhabi, United Arab Emirates; 4 Pfizer Gulf FZ LLC, Dubai, United Arab Emirates; 5 Ministry of Health and Prevention (MOHAP), Dubai, United Arab Emirates; 6 Al Ain Pharmacy Group, Al Ain, Abu Dhabi, United Arab Emirates

**Keywords:** community pharmacists, pharmacy-based vaccinations, readiness, cross-sectional study, United Arab Emirates

## Abstract

**Background:**

Pharmacist-administered vaccination is currently implemented in many countries worldwide. It has contributed to increased vaccine access and vaccine uptake. This observational cross-sectional study assessed community pharmacists’ willingness, and readiness to administer vaccines to the public in the United Arab Emirates (UAE) and relate it to national and international policies on vaccination.

**Methods:**

This research was an online survey of 24-questions that was made available to community pharmacists via social media and WhatsApp. The survey was open for six weeks (from April to June 2022). Descriptive and inferential analysis was performed.

**Results:**

The questionnaire was completed by 374 of 575 (65%) respondents. More than half (64.2%) of the respondents agreed or strongly agreed that pharmacists should be able to vaccinate and 68.4% responded that they were willing to administer vaccines if local regulations allowed them to vaccinate. Most (81.8%) expressed willingness to complete training required to be able to administer vaccines in their pharmacies. Logistic regression showed that pharmacists defined as having high readiness were significantly more willing to undergo all essential training to start a vaccination service in their pharmacies than were pharmacists with poor readiness (OR 2.647; 95% CI: 1.518–4.615; p=0.001). High readiness was also significantly associated with agreement on safety of pharmacy-based vaccination (p=0.027).

**Conclusions:**

The majority of community pharmacists surveyed showed readiness to commence pharmacy-based vaccination services. Those with high readiness characteristics are amenable to receiving essential training and consider that vaccination in the community pharmacy setting would be safe.

## Introduction

Pharmacists are amongst the most accessible and trusted healthcare professionals in the community. ^1^^–^^3^ Community pharmacists are highly trained but possibly underutilised public health professionals. ^4^ Pharmacists in the UAE do mostly dispensing of medications as their main function. Providing additional services like vaccination, pharmacist-led clinics, or other clinical services are not common in the UAE community pharmacy sector as per our knowledge.

The coronavirus 2019 (COVID-19) pandemic exposed challenges in the provision of some healthcare services, including vaccination ^5^^,^^6^; yet also highlighted the ability of community pharmacists to remain accessible to the public and provide essential services despite unprecedented demand. ^2^^,^^5^ Allowing pharmacy-based vaccination is a healthcare strategy that many countries followed to enhance access and thus increase vaccination uptake in the community. ^7^

Community pharmacies are one of the first points of contact for ambulatory patients and members of the public with relatively mild health concerns, ^8^ especially as they are conveniently located and offer extended working hours. ^5^^,^^9^ By virtue of their accessibility and reach in local communities, community pharmacists are ideally positioned to promote and provide vaccination services. ^10^ Although vaccine administration is not yet a service provided by the majority of community pharmacists in the United Arab Emirates (UAE), studies from Jordan and Saudi Arabia suggest that community pharmacists in the region have shown great willingness to deliver pharmacy-based vaccination services. ^9^^,^^11^^,^^12^

As many international studies and regional studies showed willingness of pharmacists to deliver pharmacy-based vaccination services, we decided to conduct a similar study in the UAE. This maybe an initial study in the country as similar studies were not conducted in the UAE. As the vaccination service by pharmacist started in a few pharmacies in the UAE, it was essential to study readiness and willingness of pharmacists in a national level to inform different stakeholders in the country and abroad on this proven initiate as shown in the studies above.

Considering the international and regional shifts towards pharmacy-based vaccinations, the objectives of this study were: i) to assess the readiness of community pharmacies in the UAE to start pharmacy-based vaccination services; ii) analyse if community pharmacists are willing to undertake relevant training; and iii) explore factors influencing pharmacists’ initiation of community-based vaccination.

### Aim

This study has its origins in an ongoing project to advance pharmacist-provided vaccination services in the UAE, which is being led by a working group of professionals from academia, the pharmaceutical industry, pharmacy practice and regulation, and professional societies in the UAE. The project consists of three phases: research, advocacy, and dissemination of new services. This study is a product of the research phase of the project, which involves generating an evidence base to support advocacy efforts in obtaining permission for some pharmacists to administer COVID-19 vaccination in the UAE. Aim of this research was to assess community pharmacists’ willingness and readiness to administer vaccines to the public in the United Arab Emirates (UAE) and relate it to the policy factors nationally and internationally.

## Methods

### Research design

This observational cross-sectional study surveyed licensed pharmacists practicing in community pharmacies in the UAE. The survey consisted of 24 questions grouped into eight categories: i) demographics; ii) employment details; iii) patient demand at the pharmacy; iv) interest and willingness to vaccinate at the pharmacy; v) willingness to receive training to administer vaccines; vi) perception toward vaccination; vii) factors influencing the decision to administer vaccines; and viii) perceived readiness of pharmacy to administer vaccines.

The questions were adopted and adapted from a recent survey of Jordanian community pharmacists’ readiness to provide vaccination services. ^11^ The questions were content validated by members of the expert group. A full set of validation and reliability tests of the survey instrument was not performed as it is largely same as the previously tested Jordanian study, which infact is similar to other similar studies elsewhere. This paper was written using the format provided by STROBE statements for cross-sectional studies. ^13^

### Study setting

The study setting was community pharmacies in the UAE. It was an online study.

### Paper context

Pharmacists are administering many vaccines through their pharmacies around the world, and the number of such services is increasing. When this study started in early 2022, pharmacists were not allowed to vaccinate the public in their pharmacies in the UAE. The authors of this paper are from multiple backgrounds and conducted this research and advocacy efforts to initiate pharmacist-administered vaccination in the UAE. As a result, one of the authors of this paper, who owns a chain pharmacy group in the UAE, became one of the first providers of pharmacist-administered vaccination. At the time of submission of this paper on February 2023, 14 pharmacies in the UAE were administering flu or COVID-19 vaccines to the public: https://www.doh.gov.ae/en/Abu-Dhabi-pharmacies. This paper is part of a critical change that happened in the public health of the UAE and a model to many other countries globally where pharmacists are yet to vaccinate people.

### Study population and data collection

Only responders who completed all questions were included in the analysis. The self-administered survey was created using the Survey Monkey online platform and distributed across networks of community pharmacists in the UAE. The online survey link was sent via email and WhatsApp messages. The survey was open from 22 April to 3 June 2022 (42 days). Those eligible were community pharmacists practicing in UAE who could read English and gave consent to take part in the survey. Other professions, and those who were practicing outside the country or pharmacy undergraduate students who filled the surveys were excluded.

The 11 readiness domain of the survey had the following items; vaccination specific place/room, refrigerator specific for vaccines, temperature monitor, portable refrigerator in case of power failure, anaphylaxis response kit, anaphylaxis management poster/guidance, safety box, medical waste bin, materials for hand sanitization and surface cleaning, means to store patients’ records, and access to nearby hospital if patient needs to be referred.

The seven agreement domain of the survey had the following items; pharmacists should be able to vaccinate patients, pharmacy-based vaccination would be safe, patients would prefer to get their vaccines at the pharmacy to save time, patients would trust the pharmacist to vaccinate them, pharmacists should be trained on how to vaccinate patients, pharmacists should encourage patients to get vaccinated, and pharmacists should be fully equipped to administer vaccinations.

### Sample size

Based on the Federal Competitiveness and Statistics Authority (FCSA), 8,469 pharmacists were working the health sector in 2018. ^14^ Considering the potential changes in more pharmacies being opened in the country due to COVID-19, the study investigators rounded up the pharmacist population to be 10,000. With 95% confidence interval (CI), 5% margin of error, and an effect size of 55% pharmacists being amenable to providing vaccination services in the future (derived from the recent Jordanian survey, ^11^ a sample size of 370 was calculated. A non-probabilistic sampling strategy of convenience sampling was used by disseminating survey through email and WhatsApp groups.

### Ethical considerations

The Institutional Review Board of the Gulf Medical University (Academic Health Center), Ajman, United Arab Emirates, reviewed and approved this study (Ref. no. IRB/COP/FAC/55/March-2022, dated March 23, 2022). The Review Board reviewed to ensure potential bias with the study would be minimal and the participants’ confidentiality and consent were reviewed for their compliance with good clinical practice guidelines. Written informed consent was obtained for participating in the study.

### Data analysis

Survey responses were collated and processed using a Microsoft Excel ^®^ version 16.70 spreadsheet. The following categorisation was performed to meet the assumptions of statistical tests:

• The **Readiness** domain included 11 items with ratings ranging from 0–1. Therefore, the scores ranged from 0 to 11. Hence, the total readiness score was grouped into <5.5 (poor readiness) and ≥5.5 (high readiness); 5.5 being the mid point.
• The **Agreement** domain included seven items with ratings ranging from 0–4. Therefore, the total scores ranged from 0 to 28. Hence, the total agreement score was grouped into <14 (poor agreement) and ≥14 (better agreement); 14 being the mid point. For individual agreement items, strongly agree and agree were combined as agreement; strongly disagree and disagree were combined as disagreement. Responses as neutral were removed from the analysis.

IBM ^®^ SPSS ^®^ Statistics Version 26 Armonk, NY was used to perform Fisher's Exact Test, Pearson Chi-squared test, and logistics regression. The Mann-Whitney U test was used to find statistical significance at p-value 0.05.

## Results

The calculated sample size was met in 42 days with 374 quality-checked responses being analysed for the results from a total of 575 pharmacists that responded (i.e., 65% response rate). ^13^ Of the 201 respondents not included in the analysis, 186 gave consent but did not finish the survey (32%) while 15 did not agree to the consent form (3%).

Two respondents who completed the survey entered erroneous ages in the demographics questions category. These data were excluded when calculating the mean age, as they were considered to be data entry errors as participants were asked to type in their age. The two respondents’ answers to all other questions were included in the analysis.

The demographic characteristics of the 374 community pharmacists who completed the survey are summarised in Table 1. More than half of the respondents were female. All Emirates were represented in the survey respondents, with almost half (45.4%) being from Abu Dhabi. Over three-quarters of the respondents (78.3%) worked at or were owners of a chain community pharmacy. The majority of respondents (92.8%) stated that they worked in an urban setting, which is representative of the UAE population (2022 population statistics ^15^). A majority of the participants were BPharm qualified while others held higher PharmD or MPharm qualifications.

**Table 1.** Study population (n=374) demographics and characteristics.

Gender	198 (52.9%) Female; 176 (47.1%) Male
Age	Mean 33.2 years (SD 7.6) ^†^
Education	245 (65.5%) BPharm
	53 (14.2%) MPharm
	76 (20.3%) PharmD
Emirate	170 (45.4%) Abu Dhabi
	142 (38.0%) Northern Emirates ^¥^
	62 (16.6%) Dubai
Rural/Urban	347 (92.8%) Urban (City); 27 (7.2%) Rural (Village)
Type of Pharmacy	293 (78.3%) Chain; 81 (21.7%) Independent

^†^ Two erroneous answers were excluded.

^¥^ Consolidated data for Ajman, Fujairah, Ras Al Khaimah, Sharjah, and Umm Al Quwain.

More than half of the respondents (55%) had at least six years’ experience working as a pharmacist. Nearly all respondents (94.4%) were employees rather than owners of a pharmacy and more than half (51.6%) either worked in or owned a pharmacy that employed ≥5 people. In terms of customer facing time, almost two-thirds (63.4%) of the respondents reported that they interacted with at least 20 customers per day.

Most of the respondents (85.6%) reported encountering customers who had vaccine-related queries on a daily basis, and 11.2% of respondents provided advice, answers to questions, and/or education on vaccines and vaccination for more than half of their customers. Slightly more than half of respondents (51.8%) reported spending more than five minutes per customer providing an explanation for a vaccine-related question. Respondents answered customer vaccine-related questions about the COVID-19 and influenza vaccine most often and about pneumococci and meningococci vaccines least often.

If local regulations allowed pharmacy-based vaccinations, 68.4% of respondents were willing to administer vaccines. Nearly two-thirds (64.2%) of respondents agreed or strongly agreed that pharmacists should be able to vaccinate the public. In terms of enabling pharmacists to be able to administer vaccines, 82.1% of respondents agreed or strongly agreed that pharmacists should receive appropriate training.

Respondents considered health regulatory authority support (95.2%) and a pharmacy’s ability to accommodate vaccine administration (93.0%) as being highly influential in the decision to administer vaccines at the pharmacy. More than half of respondents (68.4%) agreed or strongly agreed that pharmacists should be remunerated for administering vaccines.

**Figure 1.**
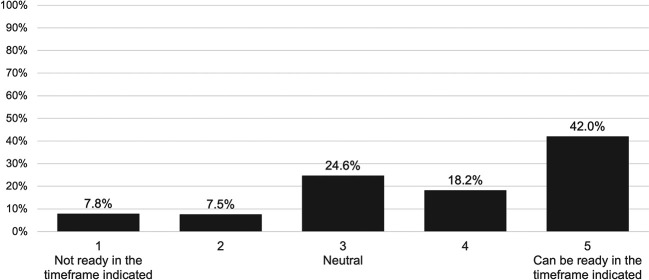
Readiness of community pharmacists in the UAE to start vaccination service in a 6-month framework.

Fewer than one in 10 pharmacists (7.8%) responded that they would not be ready to perform vaccinations within a 6- to 12-month timeframe compared with 42.0% reporting that they would be fully ready ( Figure 1).

**Figure 2.**
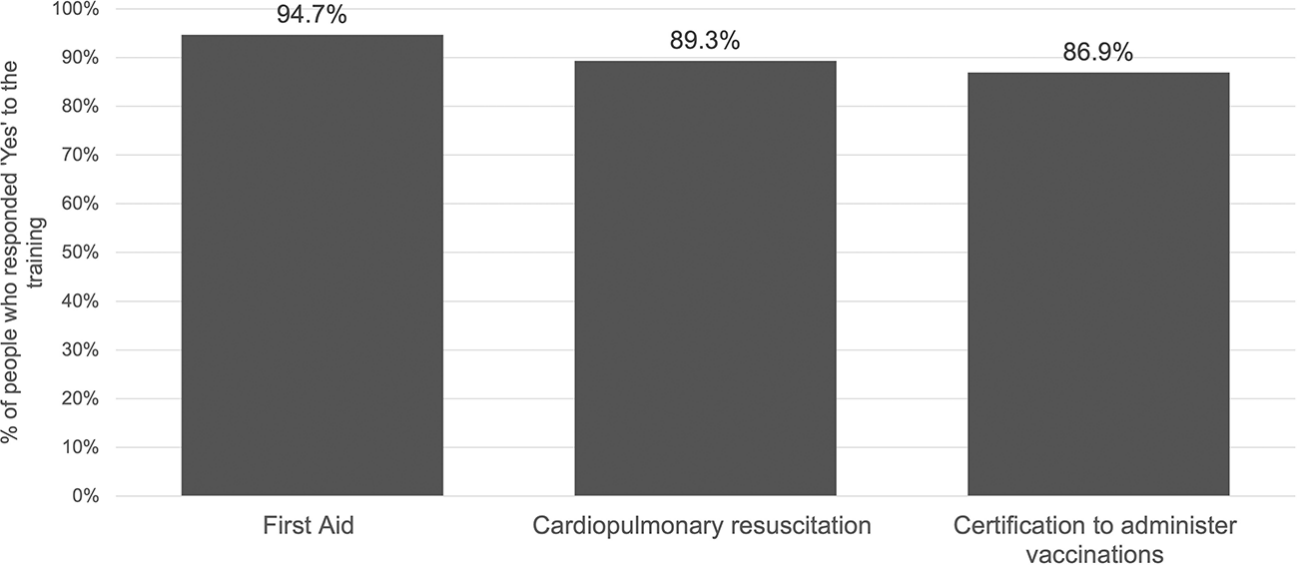
Willingness to undertake essential training.

Regarding willingness to complete training essential to be able to administer vaccines at their pharmacy, the majority of respondents responded ‘Yes’ to each of first aid training, cardiopulmonary resuscitation (CPR) training, and certification to administer vaccines. Overall, 81.8% responded ‘Yes’ to completing all three types of training and 4.0% people responded ‘No’ to all three ( Figure 2).

In terms of the association of readiness with willingness to undertake training, Fisher's Exact Test showed significant readiness by those who were willing to obtain all three essential training regarding vaccination ( Table 2).

**Table 2.** Association of readiness and willingness to start pharmacy-based vaccination.

Willingness for all essential training	Readiness		Total
	Poor readiness (Score<5.5)	High readiness (Score≥5.5)	
**Not willing at all for any training**	28 (30.4%)	40 (14.2%)	68 (18.2%)
**Willingness for all the three training (first aid, cardiopulmonary resuscitation, certification for administering vaccination)**	64 (69.6%)	242 (85.8%)	306 (81.8%)
**p-value**	Fisher’s Exact Test 0.001 (2-sided), 0.001 (1-sided). Pearson Chi-squared test 0.001 (2-sided)		374 (100%)

On further evaluation, the results of the logistic regression model demonstrated that pharmacists with high readiness (≥5.5 of the following: having space for vaccination, refrigerator specific for vaccines, temperature monitor, portable refrigerator in case of power failure, anaphylaxis response kit, anaphylaxis management literature, safety box, medical waste bin, materials for hand sanitisation and surface cleaning, means to store patients’ records, and access to nearby hospital if a patient needs to be referred) were significantly more willing to undergo all essential training (i.e., first aid, CPR, certification to administer vaccines) than were pharmacists with poor readiness (OR 2.647; 95% CI: 1.518–4.615; p=0.001).

Readiness and agreement of pharmacists that pharmacy-based vaccination would be safe ( Table 3) was significantly associated with pharmacists at high readiness compared to those at poor readiness (p=0.027).

**Table 3.** Association of readiness and agreement on safety of pharmacy-based vaccination.

Agreement: Pharmacy-based vaccination would be safe	Readiness		Total
	Poor readiness (Score<5.5)	High readiness (Score≥5.5)	
**Disagreement**	19 (28.8%)	38 (16.9%)	57 (19.6%)
**Agreement**	47 (71.2%)	187 (83.1%)	234 (80.4%)
**p-value**	Fisher’s Exact Test 0.051 (2-sided), 0.027 (1-sided). Pearson Chi-squared test 0.032 (2-sided)		291 (100%) *

* Neutral responses of 83 are removed from the total 374 ending with 291.

## Discussion

The survey shows that the UAE community pharmacy workforce is well-qualified and has a high degree of relevant customer-facing experience. From the survey responses, many of the community pharmacies in the UAE are adequately staffed and equipped to deliver vaccination services in addition to providing their traditional services. The readiness and willingness expressed by the surveyed pharmacists to vaccinate the public was promising. Collectively, these findings indicate the preparedness of community pharmacies in the UAE to provide vaccination services.

Nearly 70% of respondents supporting payment to community pharmacists for vaccination services was lower than expected. It is possible that respondents misinterpreted the question, e.g., payment to the pharmacy versus to pharmacist directly and/or due to cultural norms pharmacists may be reluctant, as healthcare professionals, to demand payment for providing vaccinations. Additionally, respondents may have believed that vaccine administration was a complementary service to selling the vaccine or that they might not be reimbursed by payers for providing the service. Low or no remuneration for additional pharmacy services could be a barrier for Such services.

The majority of the study population showing readiness to initiate pharmacy-based vaccination services in the UAE is consistent with similar studies conducted in the region. ^11^^,^^12^ In the cross-sectional survey that assessed Jordanian community pharmacists’ readiness and willingness to deliver vaccination services, 64.5% of qualified pharmacists were willing to vaccinate patients and 65.0% of unqualified participants were willing to receive the required training and qualifications to be able to vaccinate. ^11^ Another cross-sectional online survey of Jordanian community pharmacists found that that 86.6% of respondents had a high level of willingness to administer vaccines in the community pharmacy setting. ^12^

The proportion of pharmacists expressing willingness to provide vaccines services in our study and the two Jordanian studies is higher than the proportion of community pharmacists (55%) in a Saudi Arabia study who expressed willingness to vaccinate. ^9^ The difference between the studies could be explained by the Saudi Arabia survey being conducted earlier in the pandemic, when pharmacists were less certain about what was required to support public health and when they may have been more fearful of contracting COVID-19 themselves and its consequences. ^12^

Relevant training is required to equip pharmacists with the skill, knowledge, and confidence to deliver vaccination services. ^16^^,^^17^ A suitably outfitted pharmacy is also required to ensure appropriate and safe delivery of vaccination services. Necessary items include a purpose-built vaccine refrigerator, dedicated vaccination service area, general equipment necessary for vaccine administration, and an anaphylaxis response kit.

Emphasizing the importance of training, 82.5% of untrained community pharmacists in the Jordanian survey believed that pharmacists should be trained to deliver vaccination services and 80.8% of those working in unsuitably equipped pharmacies believed that community pharmacists should be sufficiently equipped to deliver vaccinations. ^11^ In reality, only 13.4% of the Jordanian pharmacists reported receiving all required training and only 8.2% reported that they worked in adequately equipped pharmacies. In our study, almost all respondents were ready to undertake specific types of training (i.e., first aid, CPR, certification) and pharmacists with high readiness characteristics were more willing to undergo all essential training to start a vaccination service in their pharmacies than were pharmacists with poor readiness.

In the Saudi Arabia survey, 67.4% of community pharmacists stated that concern about patient safety was a barrier to providing vaccination services, ^9^ and in one of the two Jordanian surveys, 40.3% of community pharmacists strongly agreed that concern about patient safety was a barrier to providing vaccination services. ^12^ Safety concerns and vaccine hesitency are barriers that need to be studied further.

In addition to receiving the relevant training, pharmacist-vaccinators need to operate in settings that are equipped to ensure the safety of patients. In one of the two Jordanian surveys, only a quarter (26.6%) of pharmacist-vaccinators reported having anaphylaxis response kits in their pharmacies and only one-fifth (20.3%) had access to anaphylaxis management literature. ^11^

In our study, pharmacists with high readiness characteristics agreed that pharmacy-based vaccinations would be safe. Moreover, the association between high pharmacist readiness and agreement on the safety of pharmacy-based vaccination was statistically significant when compared with poor readiness. In July 2022, the Department of Health (DOH) announced that community pharmacies in Abu Dhabi that have received authorisation on successful completion of a DOH-certified training course would be able to provide COVID-19 vaccination free of charge to eligible adults. ^18^^,^^19^

Evidence of the benefits of pharmacist-administered vaccination is growing internationally and in the region. Substantial evidence of the benefits of pharmacists as vaccinators is provided by a meta-analysis of 36 studies that assessed the impact of pharmacists as educators, facilitators, or administrators of vaccines on immunization rates. ^20^ All studies found an increase in vaccine coverage when pharmacists were involved compared with vaccine services provision by traditional providers. Expanding the role of the pharmacists also has the potential to reduce pressure on other healthcare providers. ^21^

It is against this background that a growing number of middle- and high-income countries have expanded the role of pharmacists to incorporate vaccination services, including the US, Australia, New Zealand, and European countries. ^8^^,^^22^ Pharmacist-administered vaccination has also been reported in low-income countries. ^23^

Vaccination is one of the most cost-effective healthcare interventions for reducing the burden of vaccine-preventable diseases. ^24^^,^^25^ Economic analyses conducted in US have demonstrated a lower mean direct cost per adult vaccination in pharmacies compared with vaccination in physician offices from the healthcare plan and patient perspectives (for zoster, pneumococcal, and influenza vaccination), ^26^ as well as from the societal perspective (for influenza vaccination). ^27^ Using pharmacies to deliver routine influenza vaccination to adults is likely to be either cost saving or relatively cost effective from a societal perspective, depending on the target population. ^27^ Moreover, administering influenza vaccines via pharmacies in addition to traditional locations during an influenza epidemic in the US was found to increase vaccination coverage, avoid up to 23.7 million influenza cases, and yield cost savings up to $US2.8 billion to third-party payers and $US99.8 billion to society. ^28^

## Conclusions

The majority of the study population demonstrated readiness and willingness to initiate pharmacy-based vaccination in the UAE. Pharmacists who agreed that pharmacy-based vaccinations would be safe was significantly associated with those who also showed readiness for starting the vaccination service. Importantly, the willingness to undertake essential training was also found among pharmacists who showed readiness. In this case, we recommend dissemination of pharmacy-based vaccination services throughout UAE.

### Strengths and limitations

The study population was considered to be a good demographic representation of community pharmacists in the UAE as a whole. It is also a strength of our study that the association of pharmacists with higher readiness and greater willingness to undergo essential training was statistically significant as was the association of pharmacists with higher readiness with agreement of the safety of pharmacy-based vaccination.

The absence of open-ended questions, which would have allowed respondents to provide greater explanation of their answers, was a limitation of the study. Also, community pharmacists not being allowed to vaccinate the public during collection of the study data might have influenced the responses of the pharmacists, though the survey mentioned ‘if approved by the ministry’.

## Data Availability

Open Science Framework: Vaccination. https://doi.org/10.17605/OSF.IO/ZQ8VC. ^28^ This project contains the following underlying data:
• Vaccination study raw data for analysis.xlsx Open Science Framework: STROBE checklist for ‘Pharmacists’ readiness and willingness to vaccinate the public in United Arab Emirates community pharmacies: A cross-sectional study’. https://doi.org/10.17605/OSF.IO/ZQ8VC. ^28^ Data are available under the terms of the Creative Commons Attribution 4.0 International license (CC-BY 4.0).
