# Supplementary material for: Pharmacists’ readiness and willingness to vaccinate the public in United Arab Emirates community pharmacies: A cross-sectional study
Source: F1000Res. 2024 Apr 22;12:292. Originally published 2023 Mar 16. [Version 2] doi: 10.12688/f1000research.131153.2 (PMC11106594; doi:10.12688/f1000research.131153.2)
Supplement: Peer review history

# Reviewer report: Reviewer response for version 2

**DOI:** 10.5256/f1000research.164896.r268406

**Published:** 2024-05-20

**Authors:** Hardika Aditama

**Affiliations:** 1 Faculty of Pharmacy, Universitas Gadjah Mada, Yogyakarta, Special Region of Yogyakarta, Indonesia

Thank you for addressing the questions. I believe the article is now ready for acceptance.

Is the work clearly and accurately presented and does it cite the current literature?

Yes

If applicable, is the statistical analysis and its interpretation appropriate?

I cannot comment. A qualified statistician is required.

Are all the source data underlying the results available to ensure full reproducibility?

Yes

Is the study design appropriate and is the work technically sound?

Yes

Are the conclusions drawn adequately supported by the results?

Yes

Are sufficient details of methods and analysis provided to allow replication by others?

Partly

Reviewer Expertise:

Social and administrative pharmacy

I confirm that I have read this submission and believe that I have an appropriate level of expertise to confirm that it is of an acceptable scientific standard.

# Reviewer report: Reviewer response for version 2

**DOI:** 10.5256/f1000research.164896.r268405

**Published:** 2024-05-03

**Authors:** Muna Barakat

**Affiliations:** 1 Applied Science Private University, Amman, Jordan

Many thanks for the authors for addressing the comments. I believe it is acceptable now.

Is the work clearly and accurately presented and does it cite the current literature?

Yes

If applicable, is the statistical analysis and its interpretation appropriate?

Yes

Are all the source data underlying the results available to ensure full reproducibility?

Yes

Is the study design appropriate and is the work technically sound?

Yes

Are the conclusions drawn adequately supported by the results?

Yes

Are sufficient details of methods and analysis provided to allow replication by others?

Yes

Reviewer Expertise:

Pharmacy and public health

I confirm that I have read this submission and believe that I have an appropriate level of expertise to confirm that it is of an acceptable scientific standard.

# Reviewer report: Reviewer response for version 1

**DOI:** 10.5256/f1000research.143966.r225189

**Published:** 2023-12-15

**Authors:** Muna Barakat

**Affiliations:** 1 Applied Science Private University, Amman, Jordan

Thank you for inviting me to review this work entitled “Pharmacists’ readiness and willingness to vaccinate the public in United Arab Emirates community pharmacies: A cross-sectional study” which aimed to assess the community pharmacists’ willingness, and readiness to administer vaccines to the public in the United Arab Emirates (UAE) and relate it to national and international policies on vaccination. 

 I believe this is very important study and will enrich the scientific background in terms of perception toward vaccines among UAE population. However, minor comments were raised and needs to be addressed

 1. Introduction section is too short, needs to be elaborated and mentioning the most important headlines covered in the title and objective, ending with the context of UAE and rationale of the study.

 2.  Methods:

- Please add inclusion and exclusion criteria.
- What do you mean by “The questions were informed in part by those of a recent survey of Jordanian community pharmacists’ readiness to provide vaccination services.11” ? did you use the same tool? Or did you adopt it? Clarify
- If you implement any modifications, did you assess the reliability and face validity?
- Regarding “The Readiness domain included 11 items with ratings ranging from 0–1. Therefore, the scores ranged from 0 to 11. Hence, the total readiness score was grouped into……….. Responses as neutral were removed from the analysis” Please elaborate more and tell the reader about score calculations and the cut-off points? Add references.
- Please add the CI for the logistic regression tables and Odds ratio
- Please add study limitations at the end of the study

Is the work clearly and accurately presented and does it cite the current literature?

Yes

If applicable, is the statistical analysis and its interpretation appropriate?

Yes

Are all the source data underlying the results available to ensure full reproducibility?

Yes

Is the study design appropriate and is the work technically sound?

Yes

Are the conclusions drawn adequately supported by the results?

Yes

Are sufficient details of methods and analysis provided to allow replication by others?

Yes

Reviewer Expertise:

Pharmacy and public health

I confirm that I have read this submission and believe that I have an appropriate level of expertise to confirm that it is of an acceptable scientific standard, however I have significant reservations, as outlined above.

# Reviewer report: Reviewer response for version 1

**DOI:** 10.5256/f1000research.143966.r194557

**Published:** 2023-09-25

**Authors:** Hardika Aditama

**Affiliations:** 1 Faculty of Pharmacy, Universitas Gadjah Mada, Yogyakarta, Special Region of Yogyakarta, Indonesia

**INTRODUCTION**

- Clarify the gaps from previous research that will be filled by this research
- What is the current role of community pharmacies in the UAE regarding vaccination?

**METHOD**

- Add information regarding the validity and reliability of the instrument used
- Mention about chi square, but does not appear in the results

**RESULT**

- Present mean with SD
- Show all demographics and characteristics in the table 1
- Show the results of logistic regression in the table
- Readiness result?

**DISCUSSION**

- Why was the 6 month framework chosen?
- Are the questions specific to a particular vaccine? What types of vaccines will be prioritized for provision?
- Based on the results of your research, what barriers are the main obstacles to immunization in pharmacies? How to deal?
- Is there a difference in readiness and willingness for pharmacists in rural-urban areas and in all characteristics/demographics?

Is the work clearly and accurately presented and does it cite the current literature?

Yes

If applicable, is the statistical analysis and its interpretation appropriate?

I cannot comment. A qualified statistician is required.

Are all the source data underlying the results available to ensure full reproducibility?

Yes

Is the study design appropriate and is the work technically sound?

Yes

Are the conclusions drawn adequately supported by the results?

Yes

Are sufficient details of methods and analysis provided to allow replication by others?

Partly

Reviewer Expertise:

Social and administrative pharmacy

I confirm that I have read this submission and believe that I have an appropriate level of expertise to confirm that it is of an acceptable scientific standard, however I have significant reservations, as outlined above.

# Response

**Published:** 2023-11-02

**Authors:** Dixon Thomas

**Affiliations:** Gulf Medical University, United Arab Emirates

Many thanks for the review comments. We believe making modifications based on this review will provide further clarity for the readers. The modifications will be performed soon.
